# Effects of guided exploration on reaching measures of auditory peripersonal space

**DOI:** 10.3389/fpsyg.2022.983189

**Published:** 2022-10-20

**Authors:** Mercedes X. Hüg, Fernando Bermejo, Fabián C. Tommasini, Ezequiel A. Di Paolo

**Affiliations:** ^1^Centro de Investigación y Transferencia en Acústica, CONICET, Universidad Tecnológica Nacional Facultad Regional Córdoba, Córdoba, Argentina; ^2^Facultad de Psicología, Universidad Nacional de Córdoba, Córdoba, Argentina; ^3^Ikerbasque, Basque Foundation for Science, Bilbao, Spain; ^4^IAS Research Center for Life, Mind and Society, University of the Basque Country, San Sebastián, Spain; ^5^Department of Informatics, University of Sussex, Brighton, United Kingdom

**Keywords:** self-generated movements, passive movements, reaching, tactile training, auditory distance perception

## Abstract

Despite the recognized importance of bodily movements in spatial audition, few studies have integrated action-based protocols with spatial hearing in the peripersonal space. Recent work shows that tactile feedback and active exploration allow participants to improve performance in auditory distance perception tasks. However, the role of the different aspects involved in the learning phase, such as voluntary control of movement, proprioceptive cues, and the possibility of self-correcting errors, is still unclear. We study the effect of guided reaching exploration on perceptual learning of auditory distance in peripersonal space. We implemented a pretest-posttest experimental design in which blindfolded participants must reach for a sound source located in this region. They were divided into three groups that were differentiated by the intermediate training phase: Guided, an experimenter guides the participant’s arm to contact the sound source; Active, the participant freely explores the space until contacting the source; and Control, without tactile feedback. The effects of exploration feedback on auditory distance perception in the peripersonal space are heterogeneous. Both the Guided and Active groups change their performance. However, participants in the Guided group tended to overestimate distances more than those in the Active group. The response error of the Guided group corresponds to a generalized calibration criterion over the entire range of reachable distances. Whereas the Active group made different adjustments for proximal and distal positions. The results suggest that guided exploration can induce changes on the boundary of the auditory reachable space. We postulate that aspects of agency such as initiation, control, and monitoring of movement, assume different degrees of involvement in both guided and active tasks, reinforcing a non-binary approach to the question of activity-passivity in perceptual learning and supporting a complex view of the phenomena involved in action-based learning.

## Introduction

The study of the role of bodily movements in spatial hearing has been receiving progressively more attention leading to an increasing understanding of how action influences auditory perception (Aytekin et al., 2008; McLachlan et al., 2021; Valzolgher et al., 2022). In the field of auditory peripersonal studies, special attention is being paid to everyday localization responses, such as reaching for an object (Farnè and Làdavas, 2002). However, few studies up to this point have integrated action-based protocols with auditory distance perception measures in peripersonal space (e.g., Rosenblum et al., 1996; Parseihian et al., 2014; Hüg et al., 2019).

In the study of perceptual skills there is a historical interest in knowing the role of bodily movements in perceptual learning. Questions such as: Are active movements, i.e., those generated by the perceiver, required for perceptual learning or do they merely facilitate it? Is it possible to improve a perceptual skill without a self-generated movement component, for example, through the proprioceptive cues present in a passive movement, i.e., assisted by a device or another person?

Existing research shows a marked tendency to assume opposing positions on these questions. Some research programs have been devoted to demonstrating that the self-generation of movements is a necessary condition for perceptual learning. Others, in contrast, challenge this claim by showing equivalences between perceptual learning or calibration in active and passive movement conditions. For example, the classic work of Held and Hein (1963) that showed the requirement of self-generated movements for kittens to develop visuospatial skills was challenged by a later replication attempt (Walk et al., 1988). In fact, many of the implications of the work by the team led by Richard Held (e.g., Held and Bossom, 1961; Held and Hein, 1963; Held and Rekosh, 1963; Held and Mikaelian, 1964), were contradicted by later replications (e.g., Pick and Hay, 1965; Singer and Day, 1966; Foley and Maynes, 1969; Baily, 1972; Gyr et al., 1979).

These discussions remain open. Some studies indicate that the training by voluntary movement improves performance in motor tasks such as moving the arm to a target compared to passive training (Lotze et al., 2003; Snapp-Childs et al., 2013). Other studies, instead, show that there are no differences in the practice of voluntary or involuntary movements on similar manual tasks (Bernardi et al., 2015), or even, that for certain movements, such as wrist supination, motor performance improves more by training with passive movements (Kwon et al., 2013).

In a recent review of Richard Held’s work, the authors sought to contribute to discussions about the role of self-generated movements in perceptual learning by studying the conceptions of activity and passivity at play in these debates (Bermejo et al., 2020). In view of the controversial experimental results, it is suggested the possibility of considering a non-dichotomous approach to the question of activity-passivity in perceptual learning. The authors proposed that what is typically taken as a clear-cut distinction between active and passive experimental conditions is in fact a spectrum of possibilities with various degrees and even different dimensions of voluntary participant involvement. These dimensions include distinct factors such as preparation for movement initiation, attempt to execute movement, movement monitoring, and movement regulation. In this way, allowing oneself to be moved by an experimenter, a situation that typically defines “passive” experimental conditions, does not entail the absence of other active elements, such as movement monitoring, active inhibition of protective reactions, and so on. This may explain why it is sometimes difficult to reach widely accepted conclusions regarding the role of self-generated activity in perception.

Hüg et al. (2019) analyzed the effects of active tactile exploration on reaching measures of auditory distance perception in the peripersonal space. The authors conducted an experiment in which participants had to estimate whether a sound source was (or not) reachable by extending the arm and estimating its distance. Compared to participants who did not receive feedback, those who could actively explore the near space and touch the sound source improved their performance. Exploration allowed them to significantly reduce the response error of the auditory perception distance and to adjust their auditory reachable space boundary. The authors of the study hypothesized that the effectiveness of the tactile feedback allowed participants to better adjust the sensorimotor loop of the reaching behavior. Participants, by learning to actively test and correct their errors, were able to calibrate their motor response according to tactile information related with acoustic and proprioceptive cues. Despite this confirmation of the important role of the feedback, it is not clear whether it is active exploration as such that facilitates perceptual learning or whether it is possible to improve performance just by giving tactile feedback associated to acoustic and proprioceptive cues but without this active component of the self-generated movement.

In the present study we extend the previous work of Hüg et al. (2019). Our aim is to characterize the kind of auditory perceptual learning in the peripersonal space without self-generated and self-executed movements, i.e., performing guided explorations. Additionally, we want to analyze the differences in learning, if any, under conditions of active and guided movements. Taking into account the discussions outlined in Bermejo et al. (2020), we denominate the group that received the “passive” training condition as the *guided* group. The term guided reflects more appropriately the complexity of the participant’s proprioceptive and subjective experience.

## Materials and methods

### Participants

Thirty adult participants (age: M = 24.9, SD = 2.31, 10 female, 29 right-handed) were recruited to participate in the study. All participants self-reported normal hearing and none had previous knowledge of the experimental setup. All participants provided written informed consent prior to the beginning of the experiment. The study was carried out in accordance with the Helsinki Declaration guidelines and the protocol was evaluated and approved by the Institutional Ethics Committee (CIEIS Hospital Nacional de Clínicas, Universidad Nacional de Córdoba, Argentina).

### Apparatus

The experimental set-up was the same as the one described in Hüg et al. (2019). The study was performed in an acoustically treated room (4.20 m long, 3.80 m wide and 2.60 m high) with walls and ceiling covered by absorbent fiberglass panels and the floor by carpet. The background noise was ∼17 dBA SPL. The room reverberation time (*T*_30_) was 170 ms at 1 kHz octave band. It was calculated by means of the deconvolution technique using REW (Room EQ Wizard) software. Both parameters were obtained with the set-up mounted but without the presence of experimenters or participants.

A wooden table covered with a sound-absorbing material (150 cm long, 30 cm wide) was placed on a support of adjustable height. The height of the table was set at 40 cm below the participant’s ears and was placed in front of them. The sound stimulus was reproduced by means of a small speaker (Sony SRS-X11; 6.1 × 6.1 × 6.1 cm) located on the table. Twenty-three target distances were labeled on a ruler fixed on the table (40–150 cm in 5 cm steps), with the origin of the scale on the vertical axis corresponding to the center of the seated participant’s head. Two small speakers located on either side of the participants emitted a masking sound that prevented them from having auditory cues about the target position changes before each trial.

The sound stimulus consisted of a train of three bursts of Gaussian broadband noise (0.02–22 kHz), 100-ms-long each one, with onset and offset ramped by 2-ms half-Hamming windows and 50 ms of silence between bursts (Macé et al., 2012; Hüg et al., 2019). The total length was 400 ms. The stimulus was calibrated to a level of 65 dBA SPL measured at the participant’s head with the sound source located at 100 cm position. The level of the sound source was fixed, i.e., it did not change throughout the experiment.

The position of the participant’s hand was captured with a motion tracker (Polhemus Patriot) placed at the center of the back of the hand, at an average distance of 15 cm from the tip of the middle finger. For participants who received guided feedback in a training phase, a sling made of a soft material was used to support and move their arm comfortably.

A software developed using MATLAB (MathWorks) allowed us to manage the experiment and the data collection.

### Design and procedure

We implemented a randomized, pretest-posttest experimental design. Each participant performed three phases in the following order: Pretest–Training–Posttest. Participants were randomly assigned to three groups: Active (AG) (10, 4 female), Guided (GG) (10, 3 female), and Control (CG) (10, 3 female). The participants remained seated on a chair located at one extreme of the table. They were blindfolded during the test and had no visual information about the experimental setup and the experimental room. An experimenter stood next to the table and placed the sound source at the target distance indicated by software on a screen. The participants had to listen to the sound stimulus with their head aligned toward the front. Once the sound stimulus ended, the participant’s task was to move the arm to rest the hand on the table at the place where the sound source was perceived (reaching response). The participants were asked to bend their torso without rising from the chair. If they perceived that the source was beyond reach, they had to report “too far” without performing any movement.

**FIGURE 1:**
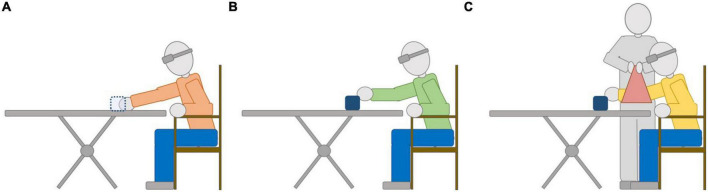
Illustration of a reaching response on the Training phase for each Group: **(A)** CG, **(B)** AG, and **(C)** GC. The sound source is depicted in dotted (or continuous) lines depending on whether it is removed (or not) by the experimenter, respectively, after the end of the sound stimulus. See text for details.

The pretest and posttest phases were the same for all groups. The experimenter removed the sound source as soon as the stimulus finished, thus none of the participants received feedback about their reaching responses. The training phase was differentiated according to each group. In the case of the CG, participants repeated the same task performed in pretest and posttest, i.e., they moved their hand to indicate their response but did not receive tactile feedback (Figure 1A). For the AG, the sound source remained on the table after the stimulus finished and participants were instructed to respond based on auditory information. If they were unable to touch the sound source on the initial reach, they could freely explore the table with their hands until they located it. If a participant reported “too far” (target perceived as unreachable) but the source was reachable, the experimenter asked them to explore until they found it (Figure 1B). For the GG, the sound source was not removed either, but in this case the experimenter moved the participant’s arm with the sling. Immediately after the stimulus ceased, the experimenter elevated their arm using the sling and moved it until their hand touched the speaker. If the source was unreachable, the experimenter maintained the participant’s arm at rest (Figure 1C).

Before starting the experiment, the participants were asked to extend their arm and hand on the table and to lean forward as much as possible without raising from the chair, in order to measure their maximum reachable distance (MRD) in centimeters.

All participants underwent a familiarization session of four trials executed at reachable and unreachable distances without feedback. In the experimental session, all distances were presented on 92 trials (23 distances × 4 repetitions) for each phase. The order of trials was randomized and balanced across participants. A short break of approximately 10 min was implemented between each phase. The average duration of the experiment without considering breaks was about 42 min.

### Data analysis

#### Data preprocessing

The trials of pre- and posttest phases were preprocessed to estimate the perceived auditory distance similarly to Hüg et al. (2019). For targets that were perceived as reachable, we obtained the measure from the motion tracker when the participant stopped the reaching movement and kept the hand still on the table (speed ∼0 cm/s). Measurements that were close to the auditory reachable space boundary were then corrected by adding the length from the tracker to the tip of the middle finger. The next step was to scale the responses with respect to the MRD of each participant, i.e., the ratio of the perceived distance and the MRD, leading to a Normalized Perceived Distance. In the same way, the Normalized Target Distance was calculated by scaling the target positions to the MRD and then rounded off to obtain intervals (bins) with a given width. The bin width was estimated based on the number of participants who contributed with responses to each bin. A maximum width of 0.035 MRD ensured data independence, since two different target distances from the same participant were not included in the same bin. A total of 33 Normalized Target Distance intervals were created (from 0.300 to 1.455 MRD, see Supplementary Table 1 for details).

Due to the nature of the task, when the speaker was perceived as unreachable (“too far” reports), the participant should not perform any reaching action, and therefore auditory distance perception responses were not collected. This situation provided partial information: the participant reported that the source was beyond reach, but it was not possible to precisely determine the perceived position. These trials (corresponding to 2% and 13% for reachable and unreachable targets, respectively) were coded assuming that the participants perceived the target at the first position beyond their MRD in the table. Supplementary Table 2 shows the percentage of trials for the different types of measurements collected in the experiment.

#### Reachability

We identified three contiguous regions of the peripersonal space, based on an estimate of the maximum reachable distances of each participant [similar to Rosenblum et al. (1996)]. A first region, closer to the participant, that we named *1-degree-of-freedom* region (1-DOF), defines the position where it was possible to reach the source only by moving the arm, without separating the back from the backrest. This region assumes a value of up to 0.55 MRD on average (M = 65.7 cm, SD = 3.7 cm; see Supplementary Table 3). A second, more distant region, called *2-degrees-of-freedom* region (2-DOF), in which the participant needed to separate the back from the backrest to reach the target. Distances in this region varied between 0.55 and 1.0 MRD (M = 118.7 cm, SD = 6.6 cm). Finally, the third region was the one that lies beyond the participant’s MRD, called the *beyond-reach* region (distances greater than 1.0 MRD).

The proportion of reaching responses for each region *p*_*ijk*_ was calculated as the total number of reaching attempts performed by participant *i*, in the phase *j*, to targets corresponding to region *k* (*k* = 1-DOF, 2-DOF, Beyond-reach), divided by the total number of trials in such phase and region. Statistical analysis was carried out employing a three-way mixed ANOVA (type III) on the reachability proportions with Group (levels: CG, AG, GG) as between-subject factor, and Phase (Pretest, Posttest) and Degree-of-freedom region (DOF) (1-DOF, 2-DOF, Beyond-reach) as within-subject factors. *Post-hoc* analysis was performed with paired and unpaired *t*-tests. The correction of Holm–Bonferroni due to multiple comparisons was applied when necessary.

#### Perceived distance

The Normalized Perceived Distance (NPD) was modeled by means of a linear mixed-effect model (LMM) using maximum likelihood with Group (between-subject factor), Phase (within-subject factor), and Normalized Target Distance (NTD, within-subject factor) as fixed effects. The model also included random effects: random intercepts by each participant and random slopes corresponding to the highest order combination of within-subject factors (i.e., Phase + NTD + Phase × NTD) (Barr, 2013). We used the *lmer* function from the *lmerTest* package (Kuznetsova et al., 2017), supplied with the R language (R Core Team, 2020), to represent the model. Its specification was:


N⁢P⁢D∼G⁢r⁢o⁢u⁢p*P⁢h⁢a⁢s⁢e*N⁢T⁢D+(P⁢h⁢a⁢s⁢e*N⁢T⁢D|P⁢a⁢r⁢t⁢i⁢c⁢i⁢p⁢a⁢n⁢t)

To assess the goodness of fit to the data we use the marginal *R*^2^ and the conditional *R*^2^ (Nakagawa and Schielzeth, 2013; Johnson, 2014). The marginal *R*^2^ considers only the variance of the fixed effects (without the random effects), while the conditional *R*^2^ takes into account both fixed and random effects. To estimate the *F*-ratios for the LMM we used an ANOVA (type III, Satterthwaite approximation of degrees-of-freedom) that provides tighter values than the χ^2^-based tests (Arnqvist, 2020).

The proportion of too-far responses was 41% (3%) out of unreachable (reachable) targets. Based on that difference, we restricted this and subsequent error analyses to responses where the Normalized Target Distances were less than or equal to 1.0, i.e., to 1- and 2-DOF regions.

#### Perceived maximum reachable distance

The peripersonal space boundary refers to the maximum distance where a person actually can reach a target and correspond to 1.0 MRD in our analyses. We measured the auditory perception of that boundary in MRD units and named it the perceived maximum reachable distance (pMRD).

The results of the above model allowed us to determine the pMRD for each participant. They were obtained by means of calculating the intersections of the LMM’s predictions with the value 1.0 of the Normalized Perceived Distance. The pMRD allowed us to infer the normalized position where participants perceived the boundary of their auditory reachable space.

#### Response errors

Signed error (SE) and unsigned error (UE) were used to measure the response error. SE was defined as *SE* = *Y* − *X*, where *X* and *Y* are the target and perceived distance in normalized form, respectively. UE was defined as the absolute value of the SE. For each trial both error measurements were calculated.

A positive value of SE is an indicator of overestimation of the distance to the target, and conversely a negative value is an indicator of underestimation of the distance. In contrast, the UE provides an overall measure of error without considering whether the distance is overestimated or underestimated.

To analyze the effect of the training phase on errors, two LMM (for SE and UE as outcome variables) similar to the one above were used. In both cases, the NTD factor was replaced by the DOF factor (levels: 1-DOF, 2-DOF). Statistical analysis was performed with ANOVA and paired and unpaired *t*-tests.

#### Change scores

To evaluate the changes that occurred due to the tactile training between the pre- and posttest phases, we calculated change scores for pMRD, SE, and UE. The change scores were obtained by averaging the repetitions under the same experimental condition for each participant and then subtracting the pretest values from the posttest values, i.e., *CS* = *S*_pos_ − *S*_pre_, where *S*_pos_ is the posttest score, *S*_pre_ is the pretest score, and *CS* is the change score.

The change score for pMRD (CS_*pMRD*_) determines whether the peripersonal space boundary was perceived to be closer (negative value), or farther away (positive value). A one-way analysis of covariance (ANCOVA, type III) was performed to examine the effects of Group on CS_*pMRD*_, after controlling for the pretest score. This score is a continuous variable representing a source of variation that was not controlled and that affects the expected value of the posttest score (Bonate, 2000; Kirk, 2012).

The change score for SE (CS_*SE*_) indicates a change in error between the pretest and the posttest phases as well as the direction of this change. If the value is positive, this indicates that the perception of distance has shifted toward more distant positions with respect to the pretest. If it is negative, it indicates that the perception of distance has shifted to closer positions. In the case of the change score for the UE (CS_*UE*_), a positive value indicates that the magnitude of errors increased from pretest to posttest, while a negative value indicates an improvement in performance (decrease in error) for distance estimation. A two-way ANCOVA was performed to examine the effects of Group and DOF regions on change score for errors (CS_*SE*_ or CS_*UE*_), after controlling for pretest score.

ANCOVA also allowed controlling what is known as regression to the mean (Galton, 1886; Yu and Chen, 2015). In addition, to reduce this effect on the measures, a previous control was performed to determine that extreme values in the pretest phase did not differ significantly across experimental groups (*p* > 0.200) (Yu and Chen, 2015).

#### Statistical software

Statistical analyses were run using R (version 3.6.3) (R Core Team, 2020) in R Studio (version 1.4.1717) (RStudio Team, 2021). For the calculation of the LMM, the ANOVA, the ANCOVA and the pairwise *t*-test we used the R-packages *lmerTest*, *emmeans*, and *rstatix* (Kuznetsova et al., 2017; Kassambara, 2021; Lenth, 2021).

## Results

### Reachability

**FIGURE 2:**
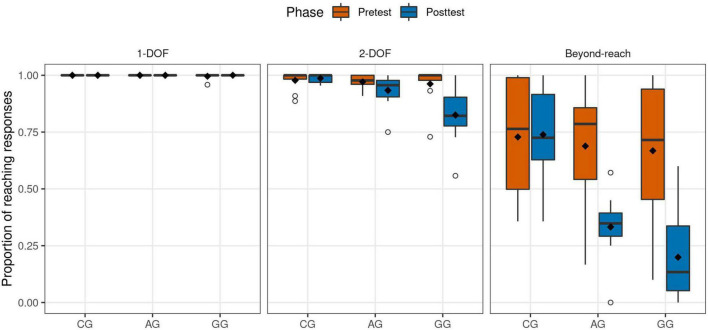
Boxplot with proportion of reaching responses according to 1-DOF, 2-DOF, and Beyond-reach regions for each group and phase. Dots represent outliers and diamonds the means.

We analyzed how the different types of responses were distributed in each region. Figure 2 shows the proportion of reaching responses according to regions and phases for each group. Values close to 1 indicate that the source was almost always perceived as reachable, while values close to 0 indicate that the sources were mostly perceived as unreachable. As can be observed, in 1-DOF, the region closest to the body, sound sources were always perceived as reachable. In 2-DOF, most of the responses continued the same pattern. In addition, in the beyond-reach region there was a substantial decrease in the proportion of attempts for GG and AG in the posttest phase A mixed ANOVA revealed that performance varied significantly as a function of the Group × Phase × DOF interaction [*F*_(2.45,33.1)_ = 7.31, *p* = 0.001].

For 1-DOF there were no significant differences. However, for 2-DOF [*F*_(2,27)_ = 4.24, *p* = 0.025], the GG participants performed a lower proportion of reaching responses in the posttest compared to the pretest [Pretest: M = 0.962, 95% CI = [0.902, 1.020]; Posttest: 0.825, [0.734, 0.916]; *t*(9) = 2.47, *p* = 0.036]. After the training phase the GG participants had also a lower proportion of attempts than the CG [*t*(9.51) = 3.99, *p* = 0.008]. These results confirm that, for the 2-DOF region, only GG participants began to perform fewer reaching responses after the training phase. This significant decrease differentiates this group from the CG participants.

In the beyond-reach region, all groups had on average a similar performance in pretest phase (CG: M = 0.728, 95% CI = [0.539, 0.917]; AG: 0.669, [0.497, 0.881]; GG: 0.668, [0.450, 0.886]). However, in posttest only the AG and GG participants had lower proportions of reaching responses (CG: M = 0.738, 95% CI = [0.585, 0.891]; AG: 0.333, [0.226, 0.440]; GG: 0.199, [0.056, 0.342]). A mixed ANOVA for this region also reported a significant effect for the Group × Phase interaction [*F*_(2,27)_ = 7.90, *p* = 0.002]. The reaching responses for AG and GG were significantly reduced in posttest [AG: *t*(9) = 3.78, *p* = 0.004; GG: *t*(9) = 4.16, *p* = 0.002], while it did not for CG. Likewise, after the training phase, the AG and GG participants had a reduction in the proportion compared to the CG participants [AG vs. CG: *t*(16.1) = 4.92, *p* < 0.001; GG vs. CG: *t*(17.9) = 5.83, *p* < 0.001]. In contrast, there were no differences between the two groups that received tactile feedback (AG and GG). These results indicate that, after the training phase, AG and GG participants ceased to perceive most of the unreachable sound sources as reachable, that is to say, they adjusted their reachability judgment appropriately.

### Perceived distance and maximum reachable distance

**FIGURE 3:**
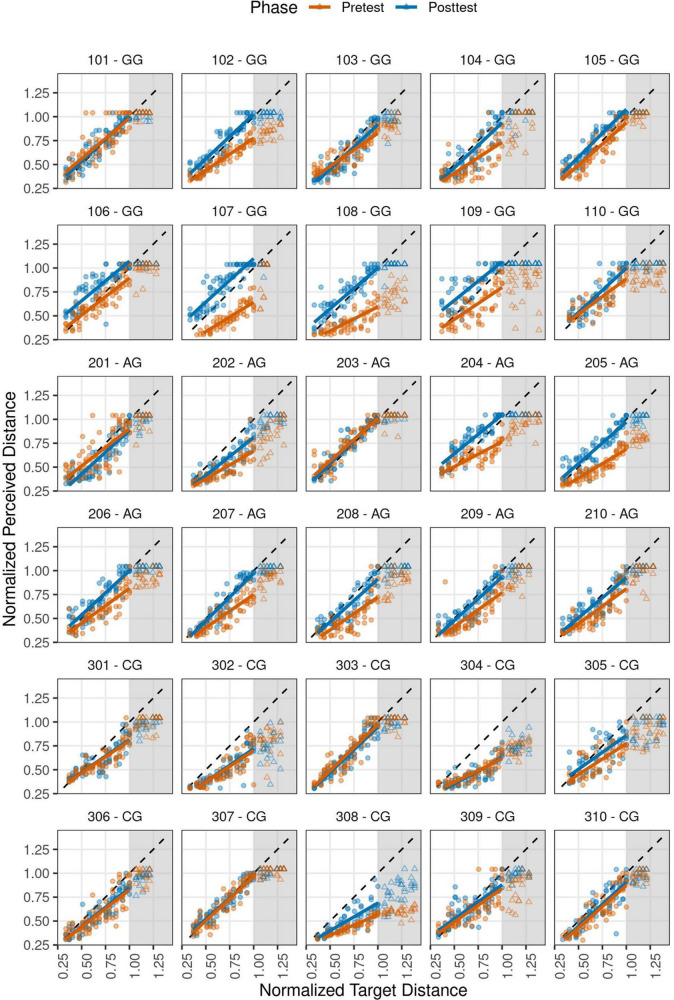
Perceived auditory distance for each participant according to each group and phase. Participants 101–110 corresponds to GG, 201–210 to AG, and 301–310 to CG. *X*-axis represents Normalized Target Distance (in MRD units) and *y*-axis the Normalized Perceived Distance (also in MRD units). The distance judgments for each trial are shown by dots for reachable target distances and by triangles for the unreachable ones. The solid lines represent the prediction of the fitted model (for reachable targets up to 1.0 MRD). Gray area indicates the unreachable zone and diagonal dashed lines the ideal performance.

Perceived auditory distance results for each participant in pre- and posttest phases are shown in Figure 3. It outlines a linear perception of the distance in the analyzed range. The regression lines were fitted by the LMM using only the reachable zone (1- and 2-DOF). The proposed model was a good fit to the data. Its total explanatory power is of 0.80 (conditional *R*^2^) and the part related to the fixed effects alone is of 0.66 (marginal *R*^2^).

In general terms, the CG participants (numbered 301–310) did not modify their response pattern after training. Their regression lines for pretest and posttest are overlapped in most cases. On the contrary, for the AG (201–210) and GG (101–110) participants, the fitted lines for each phase were different. An ANOVA found a significant effect on the interaction between Group × Phase × NTD [*F*_(2,29.5)_ = 6.64, *p* = 0.004]. This result also indicates that the perceived distance varies with target distance, phase, and training provided.

From the average regression lines (Supplementary Figure 1), we calculated the target distance at which the trend changes from overestimation to underestimation for each group and phase. For the pretest phase the obtained values, measured in MRD units, were: CG = 0.276, AG = 0.316, GG = 0.353 (the mean for pretest was 0.315 MRD, which is equivalent to ∼37 cm). While for the posttest phase these values were: CG = 0.307 (∼36 cm), AG = 0.521 (∼62 cm), GG = 1.220 (∼145 cm).

Moreover, the pMRD was calculated using the individual LMM coefficients. A pMRD value higher (lower) than 1.0 indicates that the person is overestimating (underestimating) her reachable boundary. Table 1 shows the means and 95% confidence intervals of pMRD for each group and phase, as well as of the change score. In the pretest, on average, all participants perceived the boundary in a similar distance, overestimating their MRD by ∼30%. After training, only AG and GG participants adjusted their pMRD to the one closer to actual MRD (1.0 value), i.e., their perceived boundary of peripersonal space was similar to the actual boundary.

**TABLE 1:** Mean values and change score of pMRD with 95% confidence intervals (in square brackets) according to group and phase.

Group	pMRD		Change score for pMRD
	Pretest	Posttest	
CG	1.36 [1.13, 1.58]	1.27 [1.11, 1.42]	−0.087 [−0.192, 0.018]
AG	1.33 [1.20, 1.45]	1.06 [1.00, 1.12]	−0.265 [−0.387, −0.143]
GG	1.31 [1.13, 1.49]	0.98 [0.93, 1.02]	−0.335 [−0.520, −0.150]

To analyze the size of these changes between pre- and posttest we calculated CS_*pMRD*_. An ANCOVA showed that there was a significant effect of Group [*F*_(2,26)_ = 16.8, *p* < 0.001]. AG and GG participants improved significatively their pMRD after the training phase compared to those of the CG [AG vs. CG: *t*(26) = 4.02, *p* < 0.001; GG vs. CG: *t*(26) = 5.63, *p* < 0.001].

### Response errors

#### Signed and unsigned errors

**FIGURE 4:**
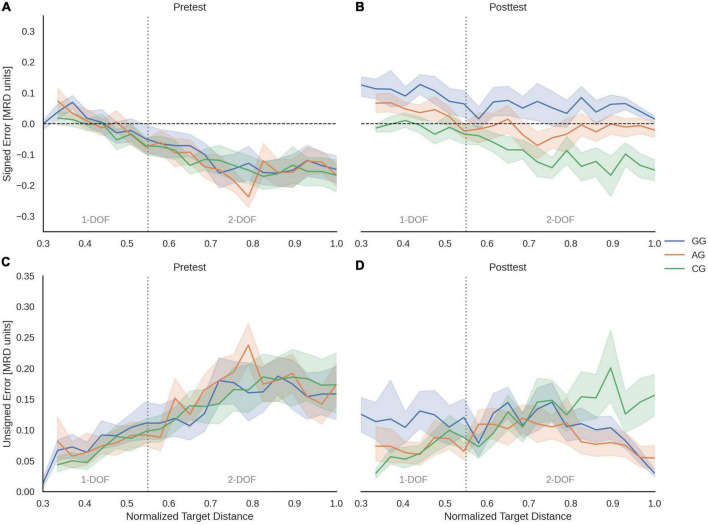
Mean of SE and UE according to Normalized Target Distances for Pretest **(A,C)** and Posttest **(B,D)** phases and each group. Color shading represents the 95% confidence intervals. Dashed black horizontal lines correspond to ideal response (no error), while dotted gray vertical lines represent the limit between 1- and 2-DOF regions.

Table 2 shows the means and 95% confidence intervals of SE according to regions, group, and phase. As can be observed, the values were mostly negative for all groups in the pretest. Figure 4A shows that the SE tended to decrease according to NTD increase. In distances close to the body, participants seemed to show a slight overestimation of the sound source’s position, while in the remaining positions, the clear tendency was to underestimate it. Statistical analysis confirmed that there were no significant differences in the pretest phase in any experimental condition. All groups performed similar underestimation errors before the training phase.

**TABLE 2:** Mean values and 95% confidence intervals (in square brackets) of SE according to regions, group, and phase.

Group	Reachable zone		1-DOF		2-DOF	
	Pretest	Posttest	Pretest	Posttest	Pretest	Posttest
CG	−0.080 [−0.129, −0.032]	−0.062 [−0.105, −0.019]	−0.021 [−0.060, 0.017]	−0.011 [−0.053, 0.031]	−0.138 [−0.200, −0.076]	−0.113 [−0.162, −0.064]
AG	−0.074 [−0.122, −0.026]	0.009 [−0.034, 0.051]	−0.006 [−0.044, 0.033]	0.037 [−0.005, 0.078]	−0.140 [−0.201, −0.078]	−0.020 [−0.069, 0.029]
GG	−0.061 [−0.109, −0.013]	0.073 [0.031, 0.116]	0.002 [−0.036, 0.041]	0.097 [0.055, 0.139]	−0.124 [−0.185, −0.062]	0.051 [0.002, 0.100]

The posttest showed an increase of overestimation errors. Both groups with tactile feedback (GG and AG) changed their previous pattern (Figure 4B). The ANOVA yielded a significant effect of Group × Phase [*F*_(2,30.2)_ = 5.22, *p* = 0.011], and Group × Phase × DOF interactions [*F*_(2,28.4)_ = 7.33, *p* = 0.003].

The *post-hoc* analysis for the reachable zone confirmed that both AG and GG modified its SE after the training phase [AG: *t*(30.1) = 3.25, *p* = 0.003; GG: *t*(30.1) = 5.29, *p* < 0.001]. There were differences between all groups [CG vs. AG: *t*(29.9) = 2.40, *p* = 0.045; CG vs. GG: *t*(29.9) = 4.60, *p* < 0.001; AG vs. GG: *t*(29.9) = 2.20, *p* = 0.045]. The AG moved closer to an error-free response, the GG went from underestimating to a persistent overestimation pattern, and the CG remained unchanged in their performance.

In the 1-DOF region, there was an effect of Group [*F*_(2,29.8)_ = 4.49, *p* = 0.020] and Phase [*F*_(1,30.2)_ = 12.2, *p* = 0.002], but not for the interaction. However, the overestimation errors were larger for GG participants in the posttest with respect to the pretest [*t*(30.1) = 3.89, *p* < 0.001]. The AG, in contrast, did not significantly modify this type of error in this region. Similarly, the GG differs from the CG after the training phase [*t*(29.8) = 3.72, *p* = 0.003], while the AG did not differ from the other two groups.

Instead, in the 2-DOF region there was a significant effect of Group × Phase [*F*_(2,30.1)_ = 7.12, *p* = 0.003]. Both groups with tactile feedback (GG and AG) changed their previous underestimation tendency [AG: *t*(30.1) = 4.22, *p* < 0.001; GG: *t*(30.1) = 6.14, *p* < 0.001]. The AG significantly reduced the SE after the training phase, maintaining a slight underestimation, while the GG changed its pattern from underestimating to overestimating the perceived distance to the source. The SE values were significantly different for GG participants with respect to AG and CG participants [AG vs. GG: *t*(30) = 2.09, *p* = 0.045; GG vs. CG: *t*(30) = 4.85, *p* < 0.001], and AG showed SE lower than CG [*t*(30) = 2.76, *p* = 0.019].

Table 3 shows the means and 95% confidence interval of UE for each group and phase, for the entire reachable zone, and for 1- and 2-DOF regions. The unsigned errors were similar in the pretest for all groups and appear to have changed in the posttest for GG at 1-DOF, as well as for GG and AG at the upper limit of 2-DOF region (Figures 4C,D). The results of ANOVA showed no significant differences for the Group × Phase and Group × Phase × DOF interactions. It is noteworthy that the GG modified their UE values after training [*t*(28.9) = 2.29, *p* = 0.029] and maintained higher UE values than the CG [*t*(29.4) = 2.62, *p* = 0.041] for distances corresponding to 1-DOF region (Figure 4D).

**TABLE 3:** Mean values and 95% confidence intervals (in square brackets) of UE according to regions, group, and phase.

Group	Reachable zone		1-DOF		2-DOF	
	Pretest	Posttest	Pretest	Posttest	Pretest	Posttest
CG	0.116 [0.091, 0.142]	0.104 [0.081, 0.126]	0.072 [0.059, 0.084]	0.070 [0.043, 0.096]	0.160 [0.115, 0.206]	0.137 [0.108, 0.167]
AG	0.122 [0.097, 0.148]	0.082 [0.060, 0.105]	0.078 [0.066, 0.091]	0.075 [0.049, 0.102]	0.167 [0.121, 0.212]	0.090 [0.060, 0.119]
GG	0.118 [0.093, 0.145]	0.109 [0.086, 0.131]	0.086 [0.074, 0.098]	0.118 [0.092, 0.144]	0.151 [0.105, 0.196]	0.100 [0.070, 0.129]

#### Change scores for errors

Change scores for SE and UE were calculated for each participant. The mean and 95% confidence interval scores according to group and region are shown in Table 4.

**TABLE 4:** Mean values and 95% confidence interval (in square brackets) of change scores for SE and UE discriminated by regions: reachable zone (1- and 2-DOF grouped), 1-DOF, and 2-DOF.

Group	Change score for SE			Change score for UE		
	Reachable zone	1-DOF	2-DOF	Reachable zone	1-DOF	2-DOF
CG	0.019 [0.008, 0.030]	0.009 [−0.006, 0.024]	0.024 [0.010, 0.038]	−0.016 [−0.024, −0.008]	−0.002 [−0.013, 0.009]	−0.023 [−0.034, −0.012]
AG	0.094 [0.077, 0.111]	0.041 [0.019, 0.063]	0.120 [0.099, 0.141]	−0.053 [−0.066, −0.040]	−0.005 [−0.021, 0.011]	−0.077 [−0.093, −0.061]
GG	0.150 [0.128, 0.172]	0.097 [0.062, 0.132]	0.176 [0.149, 0.203]	−0.026 [−0.042, −0.010]	0.028 [0.007, 0.049]	−0.051 [−0.070, −0.032]

Regarding the CS_*SE*_, it could be observed that all groups on average have shifted, in different magnitudes, their perception of the targets to more far positions. As previously stated, positive values in CS_*SE*_ indicates that perception of distance in posttest has shifted toward more distant positions with respect to the pretest. An ANCOVA confirms this observation. It showed significant differences between groups [Group: *F*_(2,495)_ = 125, *p* < 0.001] and for the Group × DOF interaction [*F*_(2,495)_ = 5.69, *p* = 0.004]. The CS_*SE*_ confirms that the GG was the group that most changed the direction of their response pattern. Considering the entire reachable zone, there was a significant change for GG compared to CG [*t*(498) = 15.4, *p* < 0.001] and to AG [*t*(498) = 7.07, *p* < 0.001]. There was also a change between AG and CG [*t*(498) = 8.38, *p* < 0.001].

These results were similar when comparing the performance of the different groups for both the 1- and 2-DOF regions [1-DOF: *F*_(2,161)_ = 26.2, *p* < 0.001; 2-DOF: *F*_(2,333)_ = 101, *p* < 0.001]. There were differences between GG and AG [1-DOF: *t*(495) = 3.84, *p* < 0.001; 2-DOF: *t*(495) = 6.16, *p* < 0.001], for GG and CG [1-DOF: *t*(495) = 6.51, *p* < 0.001; 2-DOF: *t*(495) = 14.7, *p* < 0.001] and also for AG and CG [1-DOF: *t*(495) = 2.67, *p* = 0.008; 2-DOF: *t*(495) = 8.54, *p* < 0.001]. Thus, although all participants tended to perceive the sound source farther away in the posttest than they did in the pretest, this pattern was greater in GG participants.

Regarding the CS_*UE*_, it is possible to observe in Table 4 that all groups, on average, improved their performance in distance perception (decreased their unsigned error) for the analyzed regions, except GG participants in 1-DOF region. This occurred even for CG participants, indicating that by repeating the task without tactile feedback they were able to achieve a small improvement in their performance. An ANCOVA showed significant differences for Group [*F*_(2,495)_ = 10.5, *p* < 0.001] and Group × DOF interaction [*F*_(2,495)_ = 16.7, *p* < 0.001]. For the reachable zone, there was a significant change between AG and CG [*t*(498) = 5.08, *p* < 0.001], and between GG and AG [*t*(498) = 3.68, *p* < 0.001]. However, no differences were found between GG and CG. This means that AG participants had a higher unsigned error reduction than GG and CG participants. It is worth noting that these last two groups did not differentiate their performance.

In the region closest to the participants’ body, the analysis showed that there was a main effect of Group [*F*_(2,161)_ = 12.2, *p* < 0.001]. Significant differences were found between GG and CG [*t*(495) = 3.83, *p* < 0.001] and between AG and GG [*t*(495) = 3.78, *p* < 0.001]. No differences were found between AG and CG. Thus, in 1-DOF, the improvement achieved by AG and CG participants were similar. However, both groups performed better than the GG participants.

While in 2-DOF, the AG group had a higher improvement than GG, and the latter with respect to CG participants. There was a main effect of Group [*F*_(2,333)_ = 20.3, *p* < 0.001]. Significant differences were found between AG and CG [*t*(495) = 6.42, *p* < 0.001], between GG and CG [*t*(495) = 4.47, *p* < 0.001] and between AG and GG [*t*(495) = 1.98, *p* = 0.049].

## Discussion

The study of spatial auditory abilities is increasingly taking into account multimodal interactions with nearby sound events. Our aim has been to study the effect of performing guided or active reaching exploration on perceptual learning of auditory distance in peripersonal space.

### Reaching responses on the auditory peripersonal space

The good fit of the proposed model is consistent with other studies that used linear models of auditory and visual distance perception in near-field or peripersonal space (Witt and Proffitt, 2008; Kan et al., 2009; Parseihian et al., 2014; Hüg et al., 2019). However, it should be noted that other experiments on auditory distance perception have used logarithms of target and response distances (Kim et al., 2015; Hládek et al., 2021). For the positions nearest to the body, both types of modeling seem to adjust to the data.

In conditions without tactile feedback, our results showed a general trend toward a progressive underestimation of the auditory perceived distance as the source was located further away from the participant. However, in the closest region to the body we observe a slight tendency to overestimate the position of the source, but this was reversed at 0.315 MDR on average (equivalent to ∼37 cm), from where the participants began to underestimate. A similar profile was found in the previous study of Hüg et al. (2019), as well as in other near-field auditory distance perception experiments. In its most equivalent conditions, it is possible to interpret that the transition from overestimation to underestimation occurred approximately at 40–45 cm (Hüg et al., 2019), 45–60 cm (Brungart, 1999), and 50–55 cm (Parseihian et al., 2014). Comparison with other studies of distance perception that included some close body positions is difficult due to methodological reasons, mainly related to the type of response, range of distances evaluated, the stimuli employed, and the test administration conditions (e.g., Zahorik, 2002; Fontana and Rocchesso, 2008; Kearney et al., 2012).

Distance perception performance (where is the sound source?) and reachability judgments (can I reach the target if I move my hand?) are related. Without training, the underestimation of the perceived distance increases at positions close to the boundary of reachable space. This may be related to previous research that showed evidence of overestimation in reachability judgments (Carello et al., 1989; Rosenblum et al., 1996). In our study, the perceived maximum reachability distance (pMRD) was estimated at about 1.30–1.35 MRD. Similar overestimation proportions (30%) were found in a study of visual reachability (Rochat and Wraga, 1997). One possible reason for the greater tendency to underestimate perceived distance compared to Brungart (1999) and Parseihian et al. (2014), is to consider that in our study the participant was asked to give a location response only if they estimated that they could effectively reach the source. This may have favored the adoption of a judgmental attitude that encourages underestimation.

### Effects of active and guided exploration

Regarding the effect of active training on performance, we found that AG participants were able to correct their trend of perceived auditory distance underestimation. The AG exhibited the greatest decrease in the magnitude of their errors across the full range of positions analyzed. In Hüg et al. (2019) study, it was postulated that active tactile feedback allows to improve performance by means of a particular calibration process. It consisted in adjusting the perception of the whole range of target distances from a midpoint at which participants were accurate (errors were close to zero), which entails the cost of an overestimation of prior positions and an underestimation of subsequent target distances. The present work seems to support these calibrations criteria. Participants who were able to actively explore their environment appear to have established a transition point that is located at ∼0.52 MRD (62 cm on average) and roughly coincides with the boundary between 1-DOF and 2-DOF regions.

On the other hand, GG participants were the ones that most changed the direction of their response pattern, but this did not mean an improvement in performance with respect to the other groups. After training, they made fewer reaching responses, not only in the beyond reach region, but also in the 2-DOF region. This suggests that they perceived many reachable sources as beyond of their reach capabilities. This group also exhibited significantly higher overestimation errors of perceived distance than the other groups for all reachable target positions. Despite the large change in the direction of their responses, they did not improve their performance. Rather, the analysis of change scores for unsigned errors revealed that they did not differ from the improvement made by the CG, which is produced by the mere repetition of the task.

We can postulate that guided exploration movements would generate a different type of calibration than active exploration. GG participants appear to adjust only one general fitting criterion for all the reachable range of distances, while AG participants seem to use region-specific calibration criteria for 1 and 2-DOF. It is possible to hypothesize that the GG established its transition point further than that of the AG, at ∼1.2 MRD. Prior to this point the tendency was to overestimate the source. Although it was not measured in this work, underestimation errors would be expected after this transition point.

Regarding the observed changes on pMRD, only training groups adjusted their perception to their body reachability possibilities. In AG this could be related to their good performance in 2-DOF, in which they had the smallest errors. In other words, by reducing localization errors in targets that were close to the reachability boundary, they were able to recognize it better. In GG, on the other hand, this improvement could be explained by the proximity of the boundary with the transition point mentioned before, in which the error is close to zero. It is worth noting that our results provide evidence on the possibility of modifying the peripersonal space boundary through guided exploration. This is consistent with recent studies that shows the plasticity of peripersonal space to expand or contract under diverse conditions, such as brief training, the use of tools, or limb immobilization (Noel et al., 2020; Toussaint et al., 2020).

In hypothetical terms, we postulate that the training allows participants to establish egocentric reference points from which participants learned to calibrate the auditory space in relative terms. It involves applying a criterion that generates systematic errors of underestimation or overestimation in different space regions. These reference points seem to coincide with particular postural constraints for each group. In the case of the AG, they appear to coincide with the point at which the participant has to separate the back of the chair. In the case of the GG, it would be located beyond the position that implies the maximum postural stretch. The proprioceptive cues provided by these postures associated with sound stimuli can provide reference points for more general calibration. In this sense, the present work provides support for postulates that indicate the relevance of the posture in the calibration of reaching to a sound source (Rochat and Wraga, 1997).

### Implications for active and passive sensorimotor learning

What factors could account for these differences in calibration strategies? In the GG, participants had cues about the difference between the auditory perceived position and proprioceptive information of their arm. This information could allow them to perform general sensorimotor adjustments based on the experience of discrepancy between the auditory information and the final position of the guided arm. This explanation was also used in vision studies to describe calibration in conditions where there were no active movements or feedback. For example, Mostafa et al. (2019) evaluated the ability to localize an unseen hand after it was moved actively or passively and underwent different treatments of exposure to a visual-proprioceptive discrepancy. The authors demonstrated that the localization of the hand could be similar in both movement conditions, which supports the importance that proprioception has in estimating one’s own limb position.

In addition to the proprioceptive and auditory information, the AG also had information arising from the possibility of failing to contact the source and correcting the response accordingly. There is extensive evidence that trial-and-error reinforcement is a solid resource for learning sensorimotor skills (Krakauer and Mazzoni, 2011). This group had the possibility to make fine-tuning adjustments, so they had more motor practice in the task and more information to allow them to perform a tighter calibration. In addition, it has been proposed that more effective online control of arm movement is associated with better motor planning and with less variable and more accurate reaching movement endpoints (Glazebrook et al., 2016). The performance of GG could thus be related to the adverse effects of the motor control loss in the training phase on the motor planning of the posttest.

It is worth mentioning that some GG participants, after the study, spontaneously mentioned that when they completed the training phase, they noticed that the sound source tended to be farther away than they had predicted. Therefore, they decided in the posttest phase to intentionally stretch their arm a little farther away than the site where they perceived the source. This type of decision on the adjustment criterion could contribute to the explanation of their tendency to overestimate the distance after training. Heft (1993) suggested that the under- and overestimation errors reported in reachability verbal judgments may be related to the use of an analytical or reflexive strategy. It would then be possible to hypothesize that conscious decisions such as those mentioned by some participants, could have contributed to the overestimation of distance perception bias.

Our results can also be related to others in the field of sensorimotor learning of arm and hand movements. Trewartha et al. (2015) compared spatial object localization and recognition using active and passive reaching movements. Participants, without being able to see their hand or arm, had to explore an environment until they found visual targets located in different positions that had been previously reached actively or passively (guided by a robotic device). Although the performance measures that the authors used are not directly comparable to our own, it is interesting to note that they found significantly more correct responses in the active reaching condition. In another study, Beets et al. (2012) compared passive and active motor training for learning a novel bimanual coordination task. Their results revealed that passive training achieved comparable performance to active training in aspects such as global timing of movements. However, for other indicators, such as spatiotemporal coordination of movements, active training was more effective. The authors remark that some degree of learning is possible with proprioceptive input, and that this depends on the complexity of the task. They also suggest that the better performance of the active group is a consequence of a more active participation in the processes of detection and correction of movements errors. In agreement with our study, Beets et al. could demonstrate that passive training can generate learning although not as successful as active training.

### Agency and passive learning

The differences in sensorimotor learning in the AG and GG, while noticeable, are subtle. This is due to the difficulty in neatly separating the kind and degree of participant activity involved in each condition. Bermejo et al. (2020) distinguish at least three possible dimensions of activity that can be at play in active or passive conditions: (i) *action initiation*, which involves prospective intentional aspects and is associated with impulses to begin an action as well as with a sense of urge or preparation; (ii) *action control*, which is related to the continuous regulation of movements and involves adapting to deviations or compensating for unexpected events and obstacles; and (iii) *action monitoring*, i.e., the attention to the actions carried out in order to achieve a goal. These dimensions contrast with the binary notion of activity/passivity that has been used in the past, in the sense that in each of them different aspects and different degrees of activity can come into play.

Although not actively regulating the action, a participant may still try not to resist the externally imposed movement, or accompany it, or attempt to predict what the next stage will be, all of which imply a high degree of activity. Distinguishing the degree of activity in the action monitoring dimension is even more complex. In the AG, participants were obviously attentive to reaching the sound source. In the GG they were also always aware of goal achievement, but the degree of this awareness is hard to assess. Participants’ monitoring in this group may range from close scrutiny of what is going on to a total lack of attention. Rather than being strictly passive, it seems reasonable to suggest that the improvement is due to a certain level of activity in action control (accompanying guided movements) and monitoring (attending to distance), which allowed them to integrate proprioceptive and auditory information.

The distinction between these dimensions of activity emerges from theoretical studies on the experiential dimensions of the sense of agency (Buhrmann and Di Paolo, 2017). Changes in this sense are tightly bound with changes in body schema. D’Angelo et al. (2018) suggested that body schema and peripersonal space are sensitive to the experience of control events in space through manual actions. They affirmed that intentional body movement and their expected consequences in space are linked to the sense of agency, which is implied in enlargement or contraction of the peripersonal space. Wen et al. (2015) studied the sense of agency during continuous goal action. They found that participants’ sense of agency increased with better manual performance in a computer assisted condition relative to a self-control condition. They concluded that, when the action-feedback association was uncertain, cognitive inference was dominant relative to the process of comparing predicted and perceived information in the agency judgment.

### Final remarks

The results obtained in the present study provide an interesting background on the flexibility of auditory distance judgments. While there are antecedents that show this possibility, this is mainly through the manipulation of visual information (e.g., Warren et al., 1981; Zahorik, 2001; Calcagno et al., 2012) and to a lesser extent involve tactile information (Farnè and Làdavas, 2002; Serino et al., 2007, 2011). We are unaware of research on the role of sensorimotor tasks in the modulation of auditory distance perception, as was done in this study or in its direct antecedent (Hüg et al., 2019).

An additional fact is that we found many individual differences in the performance profiles of the participants, independent of the experimental condition and phase. This is consistent with previous work, which points to significant individual discrepancies in auditory spatial localization abilities and learning (Savel, 2009; Carlile, 2014), as well as in auditory distance perception performance (Parseihian et al., 2014; Kolarik et al., 2016). It is important to highlight that the results obtained cannot be reduced to the operationalization of the types of movements without considering the degree of individual agency experienced, nor the sensory and attitudinal conditions of each participant, among other variables of interest.

All these observations, however, demand further clarification and experimental support by extending the scope of the current study. It would be important to advance in the research of individual differences for these types of learning and on the experience of the participants. To further differentiate the sensorimotor strategies of the different groups, future work should study the kinematics of reaching movements and compare changes in parameters such as trajectory and speed. Although available evidence suggests that head movements do not contribute to improved performance in auditory distance perception (Simpson and Stanton, 1973) and reachability tasks (Rosenblum et al., 1996), it would also be valuable to systematically analyze the possible interplay of head and arm movements in active exploratory strategies. Also, it has been noted that guided motor learning is difficult to transfer to other tasks (Chiyohara et al., 2020) and that its effects tend to decay relatively rapidly over time (Tays et al., 2020). Future variations of our auditory distance perception task can be specifically targeted to explore these phenomena.

## Data availability statement

The dataset presented in this study is publicly available and can be found in an online repository: https://doi.org/10.5281/zenodo.7101219.

## Ethics statement

This study that involved human participants was reviewed and approved by CIEIS Hospital Nacional de Clínicas, Universidad Nacional de Córdoba, Argentina. The participants provided their written informed consent to participate in this study.

## Author contributions

MH, FB, and FT contributed equally to the elaboration of this manuscript and wrote the first draft of the manuscript. FT developed the software, organized the database, and performed the statistical analysis. MH and FB performed the testing and collected the data. ED provided critical input and revisions. All authors contributed to the conception and design of the study, interpreted the results, and approved the submitted version.
