# Supplementary material for: Effects of guided exploration on reaching measures of auditory peripersonal space
Source: Front Psychol. 2022 Oct 20;13:983189. doi: 10.3389/fpsyg.2022.983189 (PMC9632294; doi:10.3389/fpsyg.2022.983189)
Supplement: Supplementary file 1 [file Table_1.PDF]

Supplementary Material

Supplementary Table S1. Details for each Normalized Target Distance: number of participants averaged per bin and presented trials for each group and phase.

Normalized Target Distance	CG		AG		GG	
	Number of participants	Trials	Number of participants	Trials	Number of participants	Trials
0.300	0	0	0	0	1	4
0.335	6	24	6	24	7	28
0.370	5	20	6	24	5	20
0.405	9	36	9	36	9	36
0.440	8	32	8	32	7	28
0.475	7	28	7	28	8	32
0.510	10	40	10	40	10	40
0.545	9	36	9	36	9	36
0.580	6	24	6	24	7	28
0.615	9	36	10	40	9	36
0.650	8	32	7	28	9	36
0.685	10	40	10	40	10	40
0.720	7	28	7	28	6	24
0.755	9	36	10	40	9	36
0.790	6	24	6	24	7	28
0.825	8	32	7	28	9	36
0.860	9	36	9	36	9	36
0.895	9	36	10	40	9	36
0.930	10	40	10	40	10	40
0.965	10	40	10	40	10	40
1.000	10	40	10	40	10	40
1.035	0	0	0	0	0	0
1.070	10	40	10	40	10	40
1.105	10	40	10	40	10	40
1.140	9	36	10	40	9	36
1.175	9	36	9	36	8	32
1.210	8	32	7	28	8	32
1.245	4	16	4	16	3	12
1.280	7	28	8	32	5	20
1.315	3	12	3	12	2	8
1.350	2	8	1	4	2	8
1.385	2	8	1	4	2	8
1.455	1	4	0	0	1	4

Supplementary Table S2. Percentage of trials for the different types of measurements collected in the experiment discriminated by group and phase.

		Pretest		Posttest	
		Reachable targets	Unreachable targets	Reachable targets	Unreachable targets
CG	Reaching responses	68%	23%	69%	23%
	"Too far" reports	1%	8%	0%	8%

		Pretest		Posttest	
		Reachable targets	Unreachable targets	Reachable targets	Unreachable targets
AG	Reaching responses	66%	24%	67%	12%
	"Too far" reports	1%	9%	2%	20%

		Pretest		Posttest	
		Reachable targets	Unreachable targets	Reachable targets	Unreachable targets
GG	Reaching responses	68%	22%	63%	7%
	"Too far" reports	1%	8%	7%	23%

Supplementary Table S3. Measurements in centimeters taken from each participant for boundaries of 1-degree-of-freedom (without taking off the back of the chair) and 2-degree-of-freedom (to lean forward and extend the arm on the table) regions. Note that the boundary of 2-DOF region coincides with the maximum reachable distance (MRD) of each participant.

Group	Participant	1-DOF boundary [cm]	MRD / 2-DOF boundary [cm]
Guided	101	70	125
Guided	102	65	120
Guided	103	70	125
Guided	104	65	115
Guided	105	70	125
Guided	106	65	120
Guided	107	75	135
Guided	108	65	120
Guided	109	60	110
Guided	110	60	105
Active	201	70	125
Active	202	65	115
Active	203	65	120
Active	204	60	110
Active	205	70	125
Active	206	65	115
Active	207	65	120
Active	208	65	115
Active	209	65	120
Active	210	65	120
Control	301	65	115
Control	302	65	120
Control	303	65	120
Control	304	65	120
Control	305	60	105
Control	306	70	125
Control	307	65	115
Control	308	60	110
Control	309	65	120
Control	310	70	125

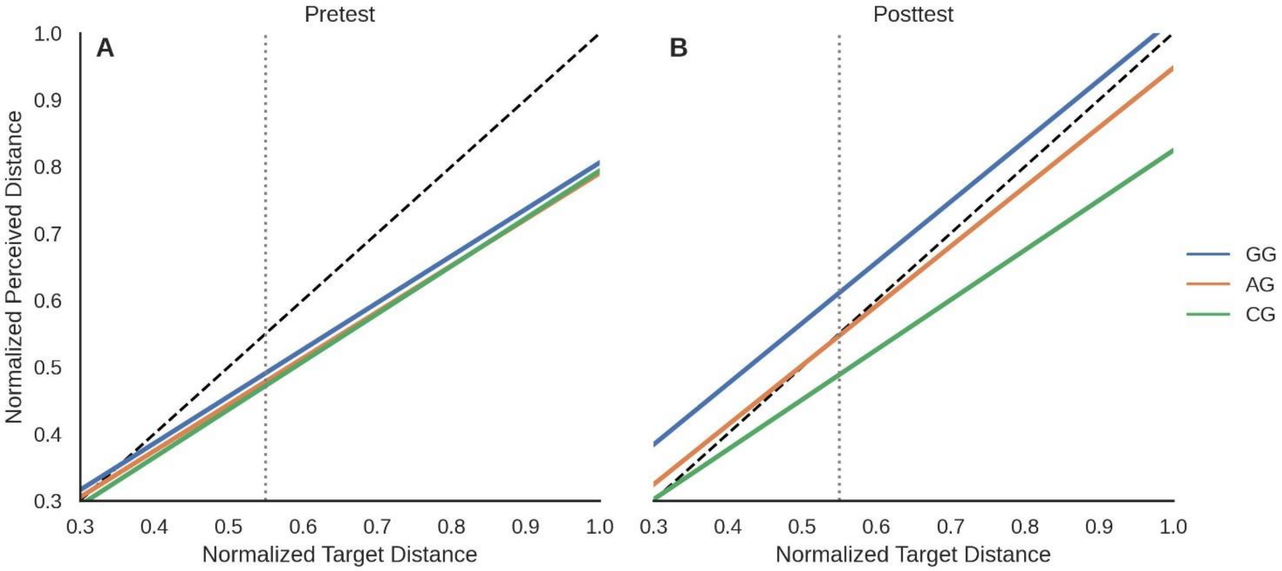

Supplementary Figure S1. Average regression lines of Normalized Perceived Distance for Pretest (A) and Posttest (B) phases. Dashed black diagonal lines correspond to ideal performance, while dotted gray vertical lines represent the limit between 1- and 2-DOF regions.
